# Hydration Is More Important Than Exogenous Carbohydrate Intake During Push-to-the-Finish Cycle Exercise in the Heat

**DOI:** 10.3389/fspor.2021.742710

**Published:** 2021-10-21

**Authors:** Craig W. Berry, S. Tony Wolf, Rachel M. Cottle, W. Larry Kenney

**Affiliations:** ^1^Noll Laboratory, Department of Kinesiology, The Pennsylvania State University, University Park, PA, United States; ^2^Graduate Program in Physiology, The Pennsylvania State University, University Park, PA, United States

**Keywords:** hydration, heat, cycling, time-trial, sports drink, dairy, carbohydrates, electrolytes

## Abstract

Dehydration ≥2% loss of body mass is associated with reductions in performance capacity, and carbohydrate (CHO)-electrolyte solutions (CES) are often recommended to prevent dehydration and provide a source of exogenous carbohydrate during exercise. It is also well established that performance capacity in the heat is diminished compared to cooler conditions, a response attributable to greater cardiovascular strain caused by high skin and core temperatures. Because hydration status, environmental conditions, and carbohydrate availability interact to influence performance capacity, we sought to determine how these factors affect push-to-the-finish cycling performance. Ten young trained cyclists exercised at a moderate intensity (2.5 W·kg^−1^) in a hot-dry condition [40°C, 20% relative humidity (RH)] until dehydration of ~2% body mass. Subjects then consumed either no fluid (NF) or enough fluid (water, WAT; Gatorade®, GAT; or GoodSport™, GS) to replace 75% of lost body mass over 30 min. After a 30-min light-intensity warm-up (1.5 W·kg^−1^) in a 35°C, 20% RH environment, subjects then completed a 120-kJ time trial (TT). TT time-to-completion, absolute power, and relative power were significantly improved in WAT (535 ± 214 s, 259 ± 99 W, 3.3 ± 0.9 W·kg^−1^), GAT (539 ± 226 s, 260 ± 110 W, 3.3 ± 1.0 W·kg^−1^), and GS (534 ± 238 s, 262 ± 105 W, 3.4 ± 1.0 W·kg^−1^) compared to NF (631 ± 310 s, 229 ± 96 W, 3.0 ± 0.9 W·kg^−1^) all (*p* < 0.01) with no differences between WAT, GAT, and GS, suggesting that hydration is more important than carbohydrate availability during exercise in the heat. A subset of four subjects returned to the laboratory to repeat the WAT, GAT, and GS treatments to determine if between-beverage differences in time-trial performance were evident with a longer TT in thermoneutral conditions. Following dehydration, the ambient conditions in the environmental chamber were reduced to 21°C and 20% RH and subjects completed a 250-kJ TT. All four subjects improved TT performance in the GS trial (919 ± 353 s, 300 ± 100 W, 3.61 ± 0.86 W·kg^−1^) compared to WAT (960 ± 376 s, 283 ± 91 W, 3.43 ± 0.83 W·kg^−1^), while three subjects improved TT performance in the GAT trial (946 ± 365 s, 293 ± 103 W, 3.60 ± 0.97 W·kg^−1^) compared to WAT, highlighting the importance of carbohydrate availability in cooler conditions as the length of a push-to-the-finish cycling task increases.

## Introduction

Physical performance capacity is dependent on a litany of factors that include hydration status, substrate availability for energy production, and the environmental conditions in which activity occurs. Aerobic exercise performance is diminished in the heat compared to cooler conditions and the mechanisms underlying this impaired performance have been reviewed (Cheuvront et al., 2010). High-intensity physical activity in the heat elicits a robust sweating response in order to maintain thermal balance which may result in significant loss of body water content if fluid is not replaced. Although this sweating response is widely variable between individuals and depends on a number of factors, including physical fitness, heat acclimation, and exercise intensity (Coyle, 2004; American College of Sports et al., 2007; Murray, 2007), it is not uncommon to observe sweat rates exceeding 1 liter per hour (Marriott, 1993). Dehydration resulting in a loss of body mass >2% has been associated with a decline in performance (Pitts et al., 1944; Adolph, 1947; Maughan and Noakes, 1991; Below et al., 1995; Sawka et al., 2001; American College of Sports et al., 2007; Maughan and Shirreffs, 2010). Additionally, during dynamic exercise in the heat, blood flow is redistributed from inactive tissues (i.e., renal and splanchnic tissues) toward active tissues (skin and skeletal muscle). However, dehydration increases competition in blood flow distribution between the skin and skeletal muscle circulatory beds to meet their thermoregulatory and energetic demands, respectively (Rowell, 1974). Concomitant cardiovascular strain (increased heart rate and reduced cardiac filling and stroke volume) attributed to increased skin blood flow limits exercise performance capacity under conditions of heat-induced dehydration (González-Alonso et al., 1998; Cheuvront et al., 2010).

Considering the highly interactive nature of exercise in the heat and dehydration, it is important to assess potential rehydration strategies that preserve and promote fluid homeostasis and how those strategies may differentially affect subsequent performance in both hot and cooler conditions. Water alone is often not sufficient for promoting rehydration, and fluids with added components are necessary to restore lost fluid. In contrast, fluids with added constituents (e.g., carbohydrate and electrolytes) may further improve hydration status (Nielsen et al., 1986; Below et al., 1995; Coyle, 2004; Jeukendrup, 2004; American College of Sports et al., 2007) and subsequent exercise performance by promoting drinking, absorption of fluid from the small intestine via activation of sodium-glucose transporters (Gisolfi et al., 2001), encouraging retention of fluid within the body and the vascular compartment (Nose et al., 1988; Shirreffs et al., 2007), and replacement of fluid and electrolytes lost *via* sweat (American College of Sports et al., 2007). The hydrating properties of these beverages are primarily a function of their electrolyte content and composition, caloric content, and total osmolality (Nielsen et al., 1986; Shirreffs et al., 1996; Maughan et al., 1997, 2016; Shirreffs and Maughan, 2000). Although sports drinks are typically prescribed to restore hydration status and improve physical performance (Wilk and Bar-Or, 1996; Rivera-Brown et al., 1999; Passe et al., 2000), more natural alternative fluid sources, such as dairy-based beverages, which have a high electrolyte concentration (Maughan et al., 1997; Shirreffs and Maughan, 2000) and similar carbohydrate and caloric concentration to traditional sports drinks (Roy, 2008), may serve as efficacious alternative fluid sources for promoting euhydration and rehydration and serve as a reliable source for exogenous carbohydrate supplementation prior to physical activity. Using the Beverage Hydration Index (BHI), Maughan et al. (2016) observed a similar hydration capacity of both skim milk and full-fat milk compared to a traditional oral rehydration solution. Our lab more recently showed an elevated BHI after consumption of a novel beverage containing protein- and fat-free milk permeate compared to both water and a traditional sports drink in well-hydrated young subjects at rest (Berry et al., 2020). This finding was mainly attributed to the increased osmolality and electrolyte concentration in the milk-permeate beverage. However, it was previously unclear how such a beverage may promote rehydration and subsequently impact physical performance following exercise- and heat-induced dehydration in young athletes, and how this beverage may compare to other common hydration sources.

The final ~10 mins of a cycling race are often considered to be the most crucial, involving dramatically increased power outputs (Menaspà et al., 2015), and placing at the finish is often decided by a few seconds. Seemingly marginal improvements in late-race performance, therefore, can substantially affect race outcomes. While a plethora of studies have compared the impact of consumption of sports drinks vs. water on performance and have been reviewed elsewhere (Shirreffs and Maughan, 2000; Shirreffs, 2009; Temesi et al., 2011; Orru et al., 2018), none to our knowledge have examined how rehydration with these distinct solutions may differentially impact performance during the important final stages of a race, i.e., the final push to the finish line after a dehydrating exercise bout. To our knowledge, no previous studies have compared the rehydration capabilities of a beverage containing ultra-filtered deproteinized milk and a traditional sports drink and their subsequent influence on performance. Likewise, whether the ambient conditions in which such an effort is performed influence beverage efficacy is unclear.

The purpose of this study was to determine how hydration status, environmental conditions, and carbohydrate availability affected selected physiological responses and performance during a push-to-the-finish task similar to that occurring during the final kilometers of a cycling race. Specifically, we sought to determine how partial rehydration with two sports drinks—a new milk permeate-based beverage (GoodSport™) and a traditional sports drink (Gatorade®)—altered push-to-the-finish time trial (TT) performance compared to water or no fluid. TT performance comparisons were made in both hot and cooler environments. We hypothesized that (1) cycling time-trial performance would be improved following rehydration with all three beverages compared to no fluid replacement, (2) consumption of both sports drinks would improve indices of hydration and TT performance compared to water, and (3) consistent with previous results from resting subjects (Berry et al., 2020), hydration status would be improved following consumption of GoodSport™ compared to water and Gatorade®.

## Materials and Methods

### Study Population

All data for this study were collected from November, 2020 to May, 2021. Ten young, well-trained (≥ 6 h/week for ≥ 6 months) cyclists (6M, 4W, 21–38 years of age) participated in studies that occurred in a hot-dry environment [35–40°C, 20% relative humidity (RH)]. All studies occurred in an environmental chamber to control these environmental conditions, and were monitored using an ASHRAE box connected to Power Lab and Laboratory Chart software (ADInstruments). A subset of four subjects who participated in that study returned to perform an additional three hydration trials in a thermoneutral (21°C, 20% RH) environment (discussed in subsequent sections). Subject characteristics for hot and thermoneutral studies are detailed in Tables 1A,B, respectively. Additional inclusion criteria consisted of the following: aged 18–45 years, body mass index <30 kg·m^2^, blood pressure <130/80 mmHg, HbA1C < 5.7%, and all premenopausal women either being eumenorrheic or taking oral contraceptives. Subjects were excluded if they reported any prior history of renal, metabolic, prostate, or cardiovascular disease or if they were taking any medications that may impact fluid balance. Fourteen subjects were enrolled in the study. Four subjects dropped out due to time commitment issues. Menstrual cycle phase was not controlled in women participating in the study, as menstrual cycle phase has been previously reported to not influence fluid replacement during or following exercise (Maughan et al., 1996). All procedures were approved in advance by the Pennsylvania State University Institutional Review Board, and all subjects gave written or verbal consent before participation in accordance with the Declaration of Helsinki. All testing was conducted in Noll Laboratory at the Pennsylvania State University.

**Table 1:** Subject characteristics for (A) hot-dry condition and (B) thermoneutral condition.

	**Mean**	**SD**	**Range**
**(A) Hot-dry condition**			
*n* (M/W)	10 (6/4)		
Age (years)	30	6	21–38
Weight (kg)	77.1	14.8	53.6–97.5
BMI (kg·m^−2^)	24.5	2.9	21.5–29.9
Systolic BP (mmHg)	118	12	102–142
Diastolic BP (mmHg)	75	8	62–88
HR (beats·min^−1^)	58	14	44–84
HbA1C (%)	4.9	0.2	4.6–5.1
**(B) Thermoneutral condition**			
*n* (M/W)	4 (3/1)		
Age (years)	31	7	22–37
Weight (kg)	83.6	17.6	59.5–97.5
BMI (kg·m^−2^)	25.4	1.9	23.8–28.2
Systolic BP (mmHg)	124	16	104–142
Diastolic BP (mmHg)	80	7	72–88
HR (beats·min^−1^)	62	13	52–80
HbA1C (%)	4.9	0.2	4.6–5.1

*Values displayed as mean, SD, and range were obtained during the pre-study screening visit. BMI, body mass index; BP, blood pressure; HR, heart rate, HbA1c, glycated hemoglobin*.

### Screening and Familiarization

All subjects underwent a screening visit prior to enrollment. Following informed consent, subjects completed a medical health history questionnaire and a fasted blood sample was then obtained for blood biochemistry analysis. Weight and height were measured to the nearest 0.05 kg and 0.01 m, respectively, while subjects wore cycling shorts and socks (women also wore a sports bra) without shoes. Subjects wore the same clothing ensemble for each of the four experimental visits. Heart rate (HR) and blood pressure (sphygmomanometry) were measured after subjects sat quietly in an upright position for at least 5 mins. An exercise time-trial familiarization session was conducted immediately after screening.

### Experimental Protocol

**Figure 1:**
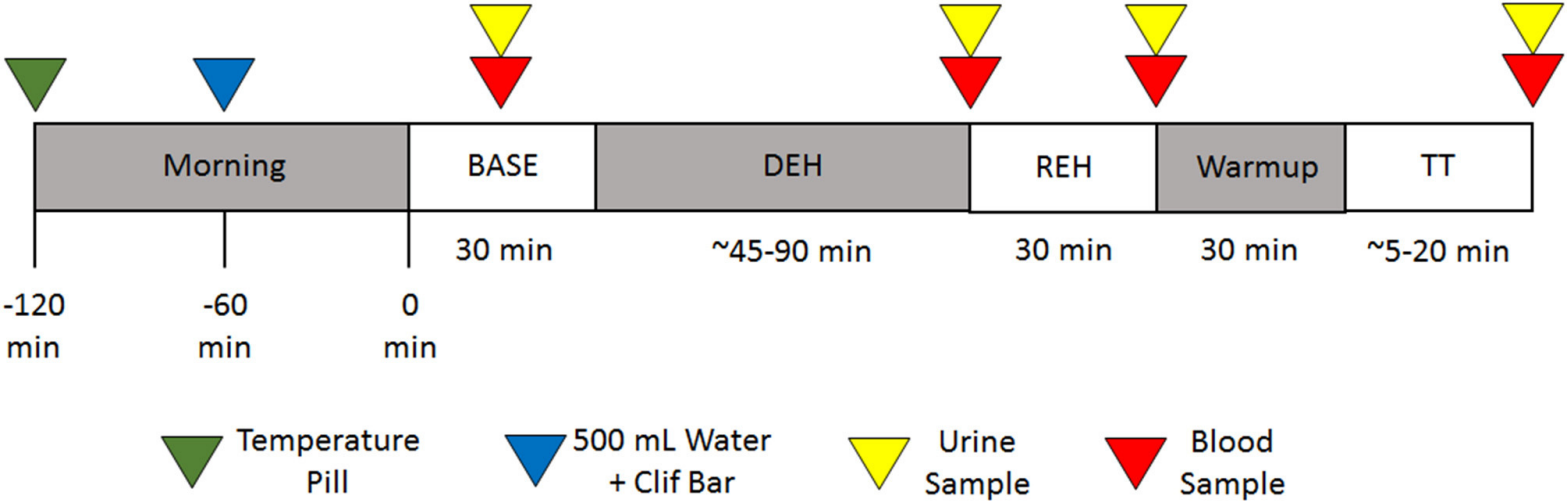
Timeline of the study.

All laboratory visits were completed in a randomized, cross-over, and counter-balanced manner. Subjects were instructed to abstain from vigorous exercise and alcohol for 24 h, caffeine for 12 h, and food ingestion for 8 h before each experiment. Additionally, subjects were asked to maintain their normal dietary and water consumption patterns for the 24 h prior to each study. A timeline for the study protocol is detailed in Figure 1. Two hours prior to each experiment, subjects ingested a temperature telemetry pill (BodyCap). One hour prior to arrival, subjects consumed 500 ml of water (Aquafina) and an energy bar (Clif Bar®: 45 g carbohydrates, 5 g fat, 9 g protein, 140 mg sodium).

All experiments occurred in an environmental chamber. Upon arrival, subjects entered the chamber, which was set at 40°C and 20% RH. During a 30-min baseline (BASE) equilibration period, a urine sample was obtained to assess urine specific gravity (USG). Body mass was then measured on a scale (Seca Scales) accurate to the nearest 0.05 kg. Subjects then sat quietly in an upright posture for the remainder of the equilibration period. At the end of the equilibration period, subjects were asked to rate their current perception of temperature (0–8; 0 = “unbearably cold,” 4 = “neutral,” 8 = “unbearably hot”) and thirst (1–9; 1 = “not thirsty at all,” 9 = “very, very thirsty”).

After completion of the equilibration period, subjects began the dehydration (DEH) period of the study. Subjects mounted their own bicycles attached to a stationary trainer (Wahoo®) and were instructed to pedal at progressively decreasing workloads until they reached a ~2% loss of body mass. The first workload was fixed for a 30-min period, with subsequent workloads fixed for shorter 15-min periods. Workload was calculated relative to body mass, with men starting at 2.5 W·kg^−1^ and incrementally decreasing by 0.25 W·kg^−1^ each period, and women starting at 2.3 W·kg^−1^ and incrementally decreasing by 0.3 W·kg^−1^ each period. Body mass was determined at the end of each stage to assess progress toward the goal of a 2% decrease in body mass.

Subjects wore a Polar chest-strap to track heart rate throughout all cycling periods. A Wahoo® phone application connected to the Wahoo® stationary trainer provided instantaneous readings of power, cadence, and speed. Subjects indicated their rating of perceived exertion (RPE) on the Borg 6–20 scale every 15 mins throughout DEH. Upon reaching the 2% loss of body mass threshold, subjects were again asked to rate their thermal and thirst perceptions. The environmental chamber temperature was then lowered to 35°C while RH remained at 20% for the remainder of the study.

Following DEH, subjects completed one of four conditions: (1) no fluid (NF), or rehydration REH with (2) water (WAT), (3) a traditional carbohydrate-electrolyte beverage (Gatorade® GAT), or (4) a novel ultra-filtered deproteinized milk (milk-permeate) beverage (GoodSport™; GS). In each trial, the amount of fluid consumed was 75% of lost body mass. This amount of fluid replacement was chosen for two reasons: (1) to reflect previously reported findings of dehydration in athletes prior to competition and typically do not voluntarily consume enough fluid to prevent onset of dehydration during competition (Arnaoutis et al., 2015; Magee et al., 2017), and (2) to prevent any potential gastric discomfort that may occur during the time trial following prior fluid replacement of 100% lost body mass. Descriptions of beverage composition and total amount of constituent ingestion for each trial are provided in Table 2. Fluid was consumed in 4 equal aliquots over a 30 min period. During the final aliquot in each of the three drinking conditions, subjects completed a sensory questionnaire for each beverage regarding beverage likability, taste, and thirst quenching properties. At the end of the 30-min REH period, measurements of blood pressure (BP), heart rate (HR), and thirst and thermal perception were obtained prior to collection of a venous blood sample. Body mass was again measured prior to collecting a urine sample.

**Table 2:** Beverage composition and total intake during hot dry condition.

	**Water**		**Gatorade** ^**®**^		**GoodSport** ^**TM**^	
	**Per liter**	**Total** **intake**	**Per liter**	**Total** **intake**	**Per liter**	**Total** **intake**
Energy (kcal)	0	0	222	313 ± 61	180	257 ± 44
Carbohydrates (g)	0	0	61	86 ± 17	64	90 ± 18
Total sugar (g)	0	0	58	82 ± 16	38	54 ± 10^*^
Sodium (mg)	0	0	444	627 ± 122	480	451 ± 88
Potassium (mg)	0	0	139	196 ± 38	1100	1571 ± 267^*^
Chloride (mg)	0	0	0	0 ± 0	980	1400 ± 238^*^
Calcium (mg)	0	0	0	0 ± 0	320	451 ± 88^*^
Magnesium (mg)	0	0	0	0 ± 0	60	85 ± 17^*^
Phosphorous (mg)	0	0	83	118 ± 23	350	494 ± 96^*^
Osmolality (mosm)	0		322 ± 2		578 ± 4	

*Values for energy, carbohydrates, total sugar, and electrolytes are label values. Osmolality was measured in quadruplicate using a freezing-point osmometer (Model 3320, Advanced Instruments, Inc., Norwood, MA, USA). Total intake is displayed as mean ± SD and was calculated from the total volume of fluid consumed over the entire study*.

* *p < 0.05 GoodSport vs. Gatorade*.

Subjects were then asked to again mount their own bicycles attached to the stationary trainer. HR, core temperature (T_c_), and power output (W) were recorded every 5 mins, and RPE every 15 mins during a 30-min warm-up period at an intensity of 1.5 W·kg^−1^. In each of the three fluid trials, a 250-ml bolus of the given fluid was provided halfway through the warm-up period and subjects were instructed to finish consuming this bolus within 10 min. The warm-up was immediately followed by a time-trial (TT) in which subjects completed 120 kJ of work as quickly as possible. Subjects were provided verbal feedback every 30 kJ throughout the time-trial. HR, T_c_, and W were recorded throughout. Upon completion of the TT, ratings of thirst, thermal sensation, and perceived exertion were obtained. Subjects then performed a 5-min cool-down at a self-selected intensity prior to a measure of body mass. To test the repeatability of the time-trial performance, 6 repeat trials were conducted over the course of the study (NF = 2, WAT = 1, GAT = 1, GS = 2).

To further examine the impact of carbohydrate supplementation on performance during a slightly longer TT in a thermoneutral environment, a subset of 4 subjects (3M, 1W) returned to the laboratory for a repeat trials of the WAT, GAT, and GS beverage conditions. The DEH, REH, and warm-up protocols were identical to the original trials; however, the TT was performed at 21°C, 20% RH. The time-trial performance goal was increased from 120 to 250 kJ. Blood and urine samples were not collected during these thermoneutral trials.

### Urine and Serum Sample Analysis

Urine samples were collected following BASE, DEH, REH, and TT in the hot-dry conditions and at BASE in the thermoneutral conditions to assess baseline hydration status. Urine mass was determined using an electronic balance accurate to the nearest 0.1 g with the mass of the empty container subtracted from the total weighed value. USG was measured at each time point using a refractometer. Urine sodium and potassium concentrations and urine osmolality were measured at each time point in triplicate (Smartlyte, Diamond Diagnostics, Massachusetts, United States) and freezing-point osmometer (Model 3320, Advanced Instruments, Inc., Massachusetts, United States, respectively).

Blood samples were collected in the hot-dry conditions following BASE, DEH, REH, and TT. These samples were collected in serum separator tubes and left in an upright position for 30 min to allow serum clotting to occur. Serum separator tubes were then centrifuged (10 min, 4°C, 4,000 rpm) and serum separated. Serum osmolality was measured immediately after centrifugation at each time point in triplicate using a freezing-point osmometer (Model 3320, Advanced Instruments, Inc., Massachusetts, United States). Hemoglobin and concentrations of hematocrit, glucose, insulin, sodium, potassium, and chloride were analyzed for each time point (Quest Diagnostics) within 2 days of sample collection.

### Calculations and Statistical Analysis

Percent change in plasma volume was calculated from hematocrit and hemoglobin concentration using the method of Dill and Costill (1974). Data collected on the Wahoo® application were downloaded and analyzed in Microsoft Excel. A two-way repeated measures ANOVA was performed to examine the effect of beverage and time on urine and serum samples. A one-way ANOVA was used to assess the effect of beverage on time-trial performance. All statistical analyses were conducted using a Tukey's HSD test for *post-hoc* pairwise comparisons. Hedge's G effect sizes were calculated and reported for select primary outcomes when comparisons were statistically different (small effect = 0.2, medium effect = 0.5, large effect = 0.8). Based on previous data demonstrating an effect size of 4.86 regarding average power output during 5-km time trial performance in either a euhydrated or hypohydrated condition (Adams et al., 2019), assuming α < 0.05, and power = 0.8, it was determined that a sample size of 8 subjects (G^*^Power) is sufficient to detect meaningful differences in power output during the time trial between different beverage trials. Data were analyzed in SAS (Cary, North Carolina, United States) using PROC MIXED model. With the exception of box plots, all data are presented as mean ± SD. Statistical significance was set a priori at α < 0.05.

## Results

### Hot Dry Condition

Baseline body mass, cardiovascular measures, glucose and insulin, and serum and urine markers of hydration status are shown in Table 3. There were no significant differences in any baseline measure across trials (all *p* > 0.05).

**Table 3:** Baseline characteristics by trial.

	**No fluid**	**Water**	**Gatorade®**	**GoodSport^**TM**^**
	**Mean ± SD**	**Mean ± SD**	**Mean ± SD**	**Mean ± SD**
Weight (kg)	77.3 ± 13.9	77.2 ± 14.0	76.8 ± 14.1	77.5 ± 14.0
Heart rate (bpm)	69 ± 16	69 ± 11	70 ± 14	68 ± 16
Mean arterial pressure (mmHg)	90 ± 8	90 ± 8	90 ± 11	90 ± 10
Glucose (mg·dL^−1^)	96 ± 13	89 ± 9	94 ± 9	93 ± 16
Insulin (mg·dL^−1^)	9.9 ± 11.3	7.5 ± 5.5	10.5 ± 8.5	11.2 ± 10.1
S_osm_ (mOsm·kg^−1^)	298 ± 3	297 ± 3	298 ± 3	298 ± 2
Serum Na^+^ (mmol·L^−1^)	139 ± 1	139 ± 1	139 ± 1	139 ± 1
U_osm_ (mOsm·kg^−1^)	298 ± 124	310 ± 226	316 ± 226	320 ± 247
Urine Na^+^ (mmol·L^−1^)	67 ± 43	62 ± 56	41 ± 37	38 ± 28
Urine specific gravity	1.009 ± 0.004	1.010 ± 0.006	1.010 ± 0.006	1.010 ± 0.006

*Values are displayed as mean ± SD. S_osm_, serum osmolality; U_osm_, urine osmolality; Na^+^, sodium; There were no significant differences in any baseline measurements among trials*.

#### Body Mass

**Figure 2:**
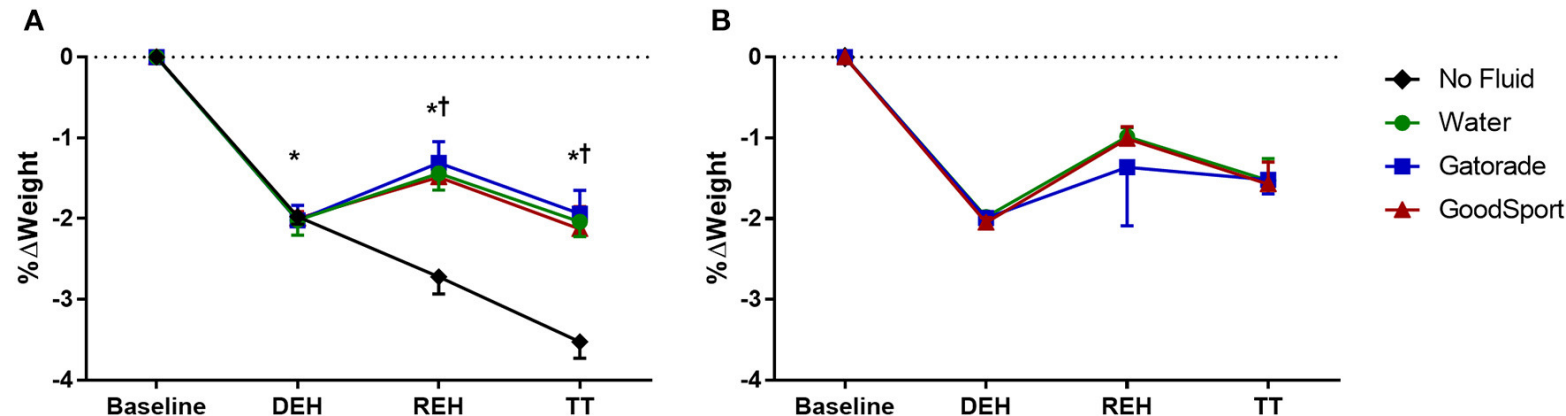
Percent change in body mass during **(A)** hot dry experiments and **(B)** thermoneutral experiments. Body mass was lower in all four trials at DEH compared to BASE in the hot-dry condition, and this persisted for the remainder of the study. Body mass was lower in NF compared to the other three conditions at REH and TT. *p < 0.05 compared to BASE. ^†^*p* < 0.05 compared to no fluid.

Percent change in body mass for the hot-dry experiment is presented in Figure 2A. As expected, body mass was similarly reduced from BASE to DEH across all four trials in the hot-dry condition (NF: −2.0 ± 0.1%; WAT: −2.0 ± 0.2%; GAT: −2.0 ± 0.2%; GS: −2.0 ± 0.1%). DEH time was not different among trials (NF: 60 ± 9 min; WAT: 63 ± 11 min; GAT: 60 ± 10 min; GS: 61 ± 9 min; all *p* > 0.34). Body mass remained lower for the remainder of each trial (all *p* < 0.0001) relative to BASE. Body mass was lower in the NF trial compared to the three drinking conditions after REH and TT (*p* < 0.0001).

#### Core Temperature (T_c_)

T_c_ was not different among conditions prior to entering the environmental chamber and did not differ among trials at any subsequent time point. T_c_ was significantly elevated following DEH and TT compared to BASE in all four conditions (all *p* < 0.0001). Following REH, T_c_ was not different compared to BASE in any of the four trials.

#### Urine Samples

There were no differences in cumulative urine output (Table 4) among the four conditions at any time point. There was a main effect of beverage (*p* < 0.04) and time (*p* = 0.006), but no interaction effect (beverage × time: *p* = 0.91) on cumulative urine output. Urine osmolality, sodium, and potassium are provided in Table 5. For urine osmolality, there was a main effect of time (*p* < 0.0001), but no effect of beverage (*p* = 0.67) or interaction effect (beverage × time: *p* = 0.93). Urine osmolality was significantly elevated compared to BASE in all four trials at both REH and TT (all *p* < 0.0001). There were no significant differences in urine osmolality among the four drink conditions at any time point. There was a main effect of beverage (*p* = 0.015) and time (*p* = 0.004), but no interaction effect (*p* = 0.83) on urine sodium; however, after adjusting for multiple comparisons, there were no differences in urine sodium among time points or beverages. There was a main effect of beverage, time, and beverage x time on urine sodium (all *p* < 0.0001). Urine potassium was higher compared to BASE in all four drink conditions at REH (all *p* < 0.02) and in NF, WAT, and GAT at TT (all *p* < 0.0001). Urine potassium was lower in GS compared to NF and WAT at both REH and TT (*p* < 0.02) and compared to GAT at TT (*p* = 0.039).

**Table 4:** Cumulative urine output and change in plasma volume.

		**Base**	**DEH**	**REH**	**TT**
Cumulative urine output (g)	No fluid	–	150 ± 66	184 ± 65	197 ± 60
	Water	–	124 ± 71	154 ± 87	170 ± 87
	Gatorade®	–	135 ± 91	151 ± 88	167 ± 91
	GoodSport^TM^	–	149 ± 126	202 ± 128	246 ± 129
ΔPlasma volume (%)	No fluid	0 ± 0	−9.0 ± 3.4^*^	−4.8 ± 2.9	−12.8 ± 3.3^*^
	Water	0 ± 0	−8.2 ± 4.4^*^	1.6 ± 2.2^†^^#^	−7.2 ± 3.2^*^^†^
	Gatorade®	0 ± 0	−9.1 ± 5.0^*^	0.0 ± 4.3^#^	−9.5 ± 5.5^*^
	GoodSport^TM^	0 ± 0	−9.3 ± 2.9^*^	−3.2 ± 2.7^#^	−7.6 ± 3.4^*^^†^

*Values are displayed as mean ± SD. Differences among beverages were assessed by two-way ANOVA. There were no differences in cumulative urine output at any time-point among trials. Plasma volume was not different among trials through REH. At TT, plasma volume was lower in NF compared to water and GoodSport*.

* *p < 0.05 vs. BASE*;

† *p < 0.05 vs. no fluid*;

# *p < 0.05 vs. DEH*.

**Table 5:** Serum and urine electrolyte concentrations and osmolality.

		**Beverage**	**Base**	**DEH**	**REH**	**TT**
Serum	Sodium	No fluid	139 ± 1	141 ± 2	141 ± 2^*^	142 ± 2^*^
		Water	139 ± 1	141 ± 1^*^	137 ± 1^†^	138 ± 2^†^
		Gatorade®	139 ± 0	141 ± 1^*^	138 ± 1^†^	140 ± 1^†^
		GoodSport^TM^	139 ± 1	141 ± 1^*^	138 ± 1^†^	139 ± 2^†^
	Potassium	No fluid	4.2 ± 0.1	4.3 ± 0.2	4.3 ± 0.2	4.2 ± 0.2
		Water	4.3 ± 0.2	4.4 ± 0.2	4.5 ± 0.2	4.1 ± 0.2
		Gatorade®	4.2 ± 0.3	4.4 ± 0.3	4.1 ± 0.2^‡^	4.0 ± 0.2
		GoodSport^TM^	4.1 ± 0.2	4.2 ± 0.2	4.8 ± 0.4^*^^†^^‡^^§^	4.4 ± 0.2^*^^‡^^§^
	Osmolality	No fluid	298 ± 3	305 ± 5^*^	302 ± 4	310 ± 6^*^
		Water	297 ± 3	305 ± 3^*^	295 ± 4^†^	301 ± 5^†^
		Gatorade®	298 ± 2	306 ± 2^*^	302 ± 2^‡^	303 ± 2^†^
		GoodSport^TM^	298 ± 2	305 ± 2^*^	305 ± 3^*^^‡^	308 ± 5^*^^‡^
Urine	Sodium	No fluid	67 ± 43	36 ± 30	65 ± 40	43 ± 32
		Water	62 ± 56	60 ± 39	85 ± 50	44 ± 36
		Gatorade®	41 ± 37	39 ± 35	63 ± 29	48 ± 30
		GoodSport^TM^	38 ± 28	35 ± 40	53 ± 32	23 ± 9
	Potassium	No fluid	29.4 ± 20.2	26.3 ± 14.3	107.5 ± 35.6^*^	109.9 ± 31.1^*^
		Water	36.2 ± 34.3	43.6 ± 33.2	113.4 ± 32.6^*^	102.6 ± 27.5^*^
		Gatorade®	26.1 ± 25.4	28.1 ± 22.3	99.8 ± 30.5^*^	86.6 ± 39.3^*^
		GoodSport^TM^	33.3 ± 41.1	36.2 ± 32.3	70.0 ± 25.1^*^^†^^‡^	52.4 ± 11.1^†^^‡^^§^
	Osmolality	No fluid	298 ± 124	231 ± 94	655 ± 169^*^	768 ± 136^*^
		Water	310 ± 226	279 ± 147	689 ± 129^*^	754 ± 138^*^
		Gatorade®	316 ± 226	254 ± 189	709 ± 85^*^	793 ± 139^*^
		GoodSport^TM^	320 ± 247	300 ± 229	681 ± 142^*^	733 ± 112^*^

*Values are displayed as mean ± SD. Differences among beverages were assessed by two-way ANOVA*.

* *p < 0.05 vs. BASE*;

† *p < 0.05 vs. no fluid*;

‡ *p < 0.05 vs. water*;

§ *p < 0.05 vs. Gatorade*.

#### Blood Samples

Percent change in plasma volume (Δ%PV) is also shown in Table 4. There was a main effect of beverage (*p* < 0.001) and time (*p* < 0.001), as well as an interaction effect of beverage × time (*p* = 0.006). Δ%PV was significantly lower in all four trials after DEH (all *p* < 0.0001). There were no differences in Δ%PV at DEH between drink conditions and Δ%PV was not different after REH compared to BASE in any drink condition. At REH, restoration of PV was significantly greater in WAT compared to NF (*p* < 0.002; *g* = 2.38), but did not differ among WAT, GAT, or GS. There was a significant expansion in PV after fluid consumption in each drink condition (all *p* < 0.004), but not in the NF condition (*p* = 0.209). At TT, Δ%PV was significantly lower compared to BASE in all four trials (*p* < 0.0002). The reduction in Δ%PV was less in WAT (*p* = 0.013; *g* = 1.65) and GS (*p* = 0.033; *g* = 1.49) compared to NF.

**Figure 3:**
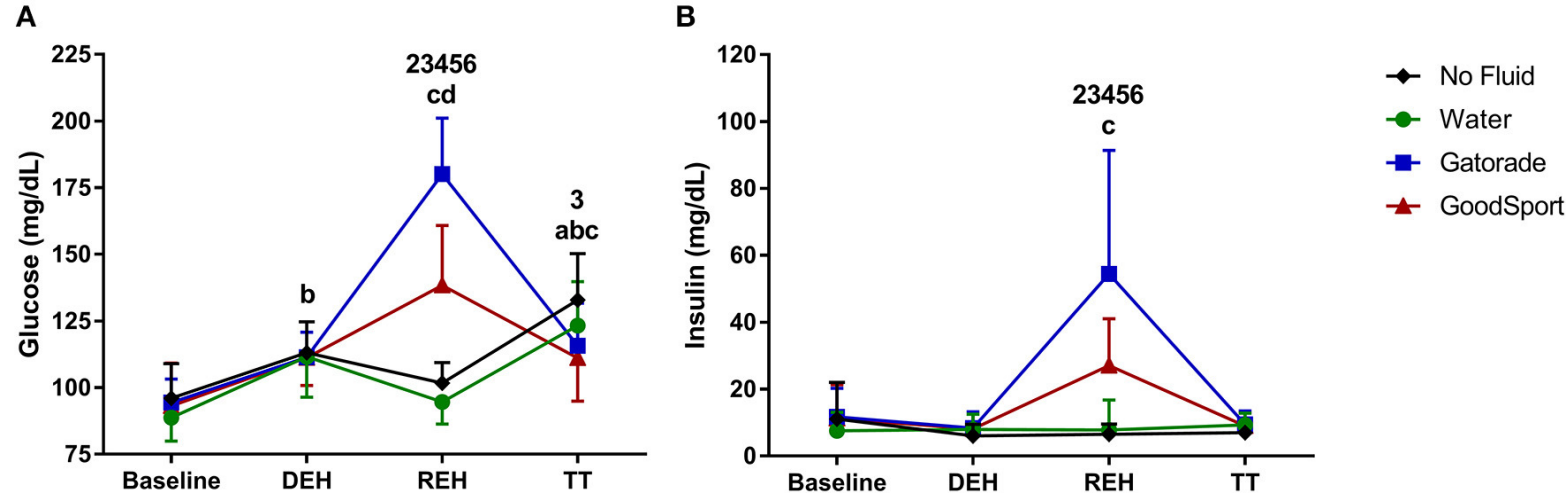
**(A)** Glucose, and **(B)** insulin concentrations. Glucose and insulin concentrations were significantly higher in Gatorade (GAT) and GoodSport (GS) at REH compared to BASE and compared to no fluid and water. The increase in glucose and insulin concentrations in GAT was higher than in GS. 1, *p* < 0.05 WAT vs. NF; 2, *p* < 0.05 GAT vs. NF; 3, *p* < 0.05 GS vs. NF; 4, *p* < 0.05 GAT vs. WAT; 5, *p* < 0.05 GS vs. WAT; 6, *p* < 0.05 GS vs. GAT; a, *p* < 0.05 NF vs. BASE; b, *p* < 0.05 WAT vs. BASE; c, *p* < 0.05 GAT vs. BASE; d, *p* < 0.05 GS vs. BASE.

Figures 3A,B display changes in glucose and insulin concentrations over time in each of the four conditions. For both glucose and insulin, there was a main effect of beverage (*p* < 0.001) and time (*p* < 0.001), as well as an interaction effect of beverage × time (*p* < 0.001). There were no differences in plasma glucose or insulin concentration among conditions at BASE or after DEH. Following REH, there was a significant increase in glucose concentration in the GAT and GS trials compared to BASE (both *p* < 0.0001), as well as compared to NF and WAT (both *p* < 0.0001). The elevation in glucose concentration in GAT was greater than in GS (*p* < 0.0001). Insulin concentration at REH was elevated in GAT compared to BASE (*p* < 0.0001). Insulin concentrations were higher at REH in GAT and GS compared to both NF and WAT (*p* < 0.002), although the increase was blunted in GS compared to in GAT (*p* < 0.001).

Serum osmolality, sodium, and potassium are shown in Table 5. There were no significant differences in serum osmolality, sodium, or potassium among conditions at BASE and after DEH. There were main effects of beverage (all *p* < 0.0001) and time (all *p* < 0.0001), as well as interaction effects of beverage × time (all *p* < 0.0001), for serum osmolality, sodium, and potassium. Serum osmolality was significantly elevated after DEH compared to BASE in all trials and remained elevated after REH compared to BASE for only GS (*p* = 0.002). Serum osmolality at REH was lower in WAT vs. NF (*p* < 0.001) and higher in both GAT and GS compared to WAT (both *p* < 0.001). After the TT, serum osmolality was significantly higher in NF and GS compared to BASE (both *p* < 0.0001). Serum osmolality at TT was significantly lower in WAT compared to both NF and GS (both *p* < 0.001) and lower in GAT compared to NF (*p* = 0.008). Serum sodium was significantly elevated in NF at REH and TT compared to BASE (both *p* < 0.03), but only at DEH in WAT, GAT, and GS (all *p* < 0.03). Serum sodium was lower in WAT, GAT, and GS compared to NF at both REH and TT (all *p* < 0.001). Serum potassium was significantly elevated in GS compared to BASE after REH and TT (both *p* < 0.03). Serum potassium was lower in GAT compared to WAT at REH (*p* = 0.03) and higher in GS compared to the three other conditions after REH (all *p* < 0.006) and compared to WAT and GAT at TT (both *p* < 0.04). Estimated glomerular filtration rate (eGFR) was significantly reduced in all four conditions beginning at DEH and remained lower for the remainder of the study (all *p* < 0.0001). There were no differences in eGFR among beverage conditions.

#### Perceptual Measures

Rating of Perceived Exertion did not differ between any condition during DEH (NF = 16 ± 3; WAT = 17 ± 2; GAT = 15 ± 3; GS = 16 ± 2) or TT (NF = 19 ± 1; WAT = 19 ± 2; GAT = 19 ± 2; GS = 19 ± 2). Thermal and thirst sensation scores (Table 6) did not differ between drink conditions at any time point. Thirst sensation was higher for NF compared to the three drinking conditions at REH (all *p* < 0.0001), and higher compared to both WAT and GAT after the TT (both *p* < 0.01), but not compared to GS (*p* = 0.13). There were also no significant differences among the three drink conditions in beverage sensory questions of overall beverage likability (all *p* < 0.79), taste (*p* > 0.46), thirst quenching (*p* > 0.49), or drinkability (*p* > 0.07). No subjects reported feeling any gastric discomfort following consumption of any of the beverages.

**Table 6:** Thermal and thirst sensation ratings.

		**BASE**	**DEH**	**REH**	**TT**
Thermal	No fluid	5 ± 0	7 ± 1^*^	4 ± 0^*^	6 ± 1^*^
	Water	5 ± 0	7 ± 1^*^	4 ± 1^*^	6 ± 1^*^
	Gatorade®	5 ± 0	7 ± 1^*^	5 ± 1^*^	6 ± 1^*^
	GoodSport^TM^	5 ± 0	6 ± 1^*^	4 ± 0^*^	6 ± 1^*^
Thirst	No fluid	3 ± 1	7 ± 2^*^	6 ± 1^*^	9 ± 0^*^
	Water	3 ± 1	8 ± 1^*^	3 ± 1^†^	7 ± 2^*^^†^
	Gatorade®	3 ± 1	8 ± 1^*^	3 ± 2^†^	7 ± 2^*^^†^
	GoodSport^TM^	3 ± 2	8 ± 1^*^	3 ± 1^†^	8 ± 1^*^

*Values are displayed as mean ± SD. Thermal sensation was based on a 0-8 scale, where 0 = “unbearably cold,” 4 = “neutral,” 8 = “unbearably hot.” Thirst sensation was based on a 1-9 scale, where 1 = “not thirsty at all” and 9 = “very, very thirsty.” Differences among beverages were assessed by two-way ANOVA. There were no differences in thermal sensation between beverages at any time-point. Thermal sensation was higher (hotter) after DEH and TT compared to BASE, and lower (cooler) after REH compared to BASE in all beverages. Thirst sensation was higher after DEH and TT compared to BASE in all beverages, and higher in no fluid (NF) after REH. Thirst sensation was lower in Water and Gatorade compared to NF after both REH and TT, but only lower in GoodSport after REH*.

* *p < 0.05 vs. BASE*.

† *p < 0.05 vs. no fluid*.

#### Time Trial Performance

**Figure 4:**
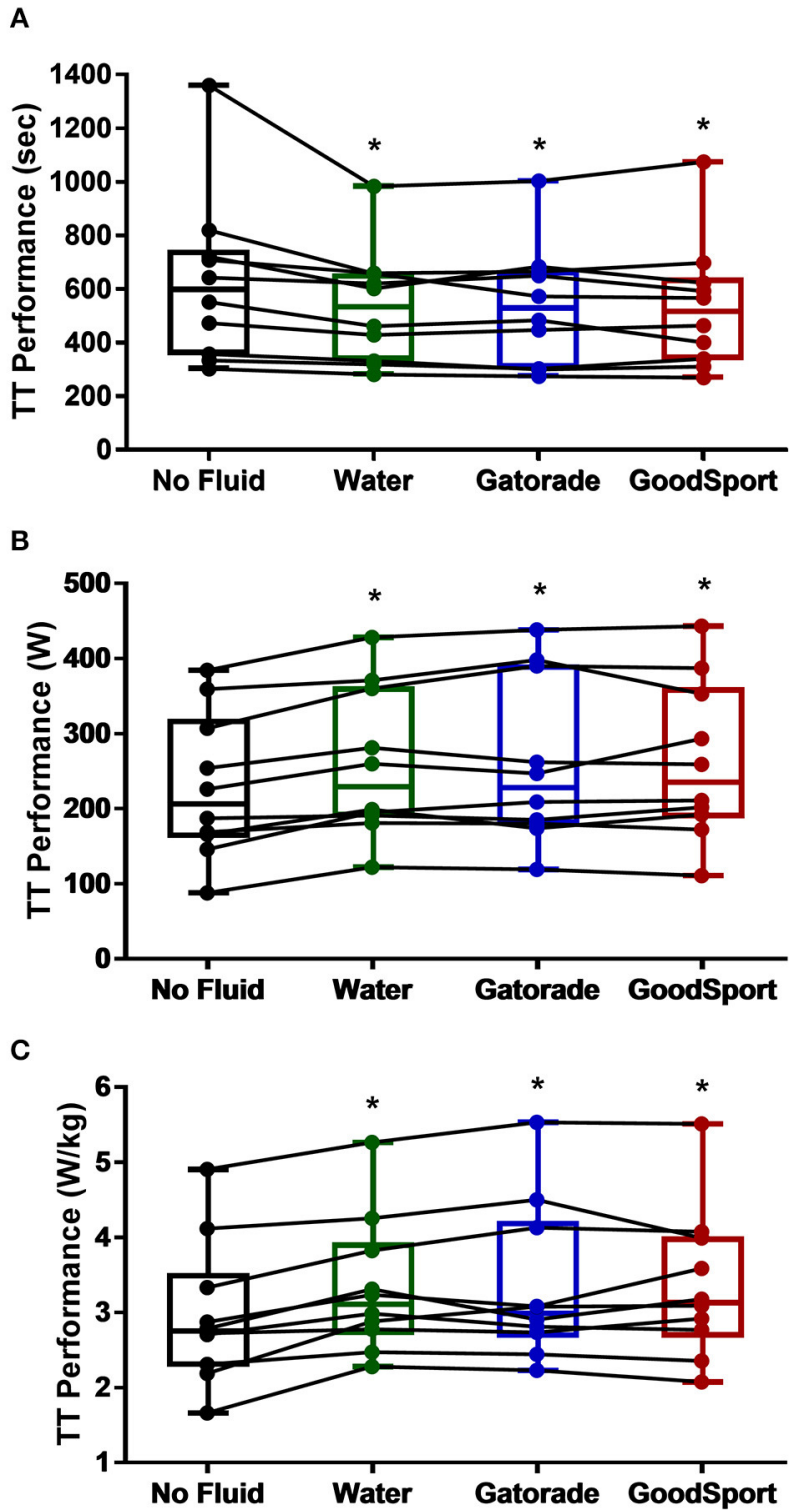
120 kJ time-trial performance in hot dry condition, measured as **(A)** time (s), **(B)** absolute power (W), and **(C)** relative power (W/kg). All three measures of time-trial performance were improved in the three beverage trials compared to no fluid. Boxes represent first and third quartiles with median values denoted by the horizontal line, while whiskers indicate minimum and maximum observations. Dots represent data points for each subject, and lines indicate changes in performance between trials for each subject. *p < 0.05 vs. no fluid.

Time-trial performance data are displayed in box-plot format in Figure 4. There was a main effect of beverage on time trial time-to-completion (*p* = 0.002), absolute power output (*p* = 0.0002), and relative power output (*p* = 0.0001). Time-trial time-to-completion (s) was significantly lower (faster) in WAT (535 ± 214 s, *p* < 0.01, g = 0.35), GAT (539 ± 226 s, *p* < 0.01, g = 0.32), and GS (534 ± 238 s, *p* < 0.01, g = 0.34) compared to NF (631 ± 310 s). Absolute power (W) was significantly higher in WAT (259 ± 99 W, *p* < 0.01, g = 0.29), GAT (260 ± 110 W, *p* < 0.01, g = 0.29), and GS (262 ± 105 W, *p* < 0.001, g = 0.31) compared to NF (229 ± 96 W). Relative power (W·kg^−1^) was significantly higher in WAT (3.3 ± 0.9 W·kg^−1^, *p* < 0.01, g = 0.32), GAT (3.3 ± 1.0 W·kg^−1^, *p* < 0.001, g = 0.30), and GS (3.4 ± 1.0 W·kg^−1^, *p* < 0.001, g = 0.40) compared to NF (3.0 ± 0.9 W·kg^−1^). There were no significant differences in time-to-completion, absolute power output, and power output relative to body mass for the time-trial among the three beverage conditions (all *p* > 0.96).

To test the repeatability of the time-trial performance, 6 repeat trials were conducted among four subjects over the course of the study as described previously and an interclass correlation coefficient (ICC) was calculated. The ICC values for time-trial performance were 0.962 for time, 0.983 for absolute power output, and 0.953 for relative power output, indicating excellent reliability/repeatability for TT performance.

### Thermoneutral Time Trials

As only four subjects completed this portion of the study, no statistical comparisons were performed. Percent change in body mass is displayed in Figure 2B. As in the hot trials, body mass was similarly reduced in each of the WAT (−2.0 ± 0.0%), GAT (−2.0 ± 0.1%), and GS (−2.0 ± 0.1%) trials following DEH and remained lower for the remainder of the study in each trial. DEH time was not different among trials (WAT: 57 ± 13 min; GAT: 54 ± 8 min; GS: 55 ± 9 min; all *p* > 0.59).

**Figure 5:**
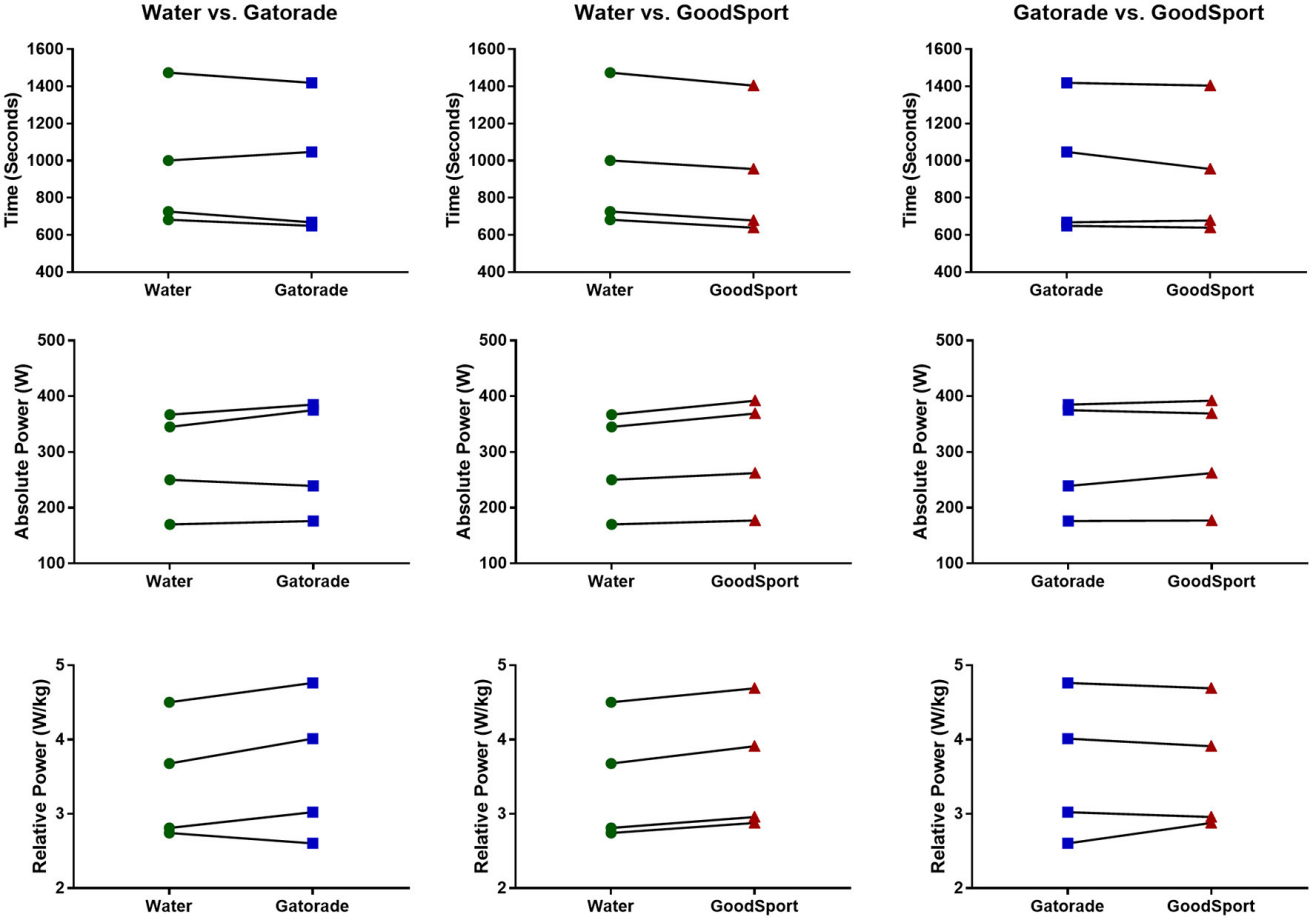
Between-beverage comparisons of 250 kJ time-trial performance in thermoneutral conditions, measured as time (s), absolute power (W), and relative power (W/kg). All three measures of time-trial performance were improved on average in both Gatorade (GAT) and GoodSport (GS) compared to water (WAT). All four subjects improved in all measures of time-trial performance in GS compared to WAT, and three of four subjects improved in GAT compared to WAT. Symbols represent data points for each subject, and lines indicate changes in performance between trials for each subject.

Fluid comparison differences for the 250 kJ time-trial performance in thermoneutral conditions are shown for each individual in Figure 5. Time-trial performance time (WAT: 960 ± 376 s; GAT: 946 ± 365 s; GS: 919 ± 353 s), absolute power (WAT: 283 ± 91 W; GAT: 293 ± 103 W; GS: 300 ± 100 W), and relative power (WAT: 3.43 ± 0.83 W·kg^−1^; GAT: 3.60 ± 0.97 W·kg^−1^; GS: 3.61 ± 0.86 W·kg^−1^) were improved in both the GS and GAT trials compared to WAT. All four subjects improved in each measure of time-trial performance in the GS trial compared to WAT, while three subjects improved in each measure of time-trial performance in the GAT trial compared to WAT.

## Discussion

The present investigation examined how hydration status, environmental conditions, and carbohydrate availability interacted to influence performance during a push-to-the-finish stationary cycling time-trial. The primary finding of this study was that in a hot, dry environment, TT time-to-completion, as well as both absolute and relative power output, were improved following rehydration compared to no fluid (NF) consumption, but were not different among drinking conditions when consuming water, a traditional carbohydrate electrolyte solution (Gatorade® GAT), or a novel sports drink containing ultra-filtered deproteinized milk (GoodSport™; GS). However, in a subset of four subjects who completed a slightly longer TT in a thermoneutral environment, performance was improved in all subjects when comparing GS to WAT and in three of four subjects when comparing GAT to WAT. These findings collectively indicate that hydration status has a greater influence on performance during relatively short-duration push-to-the-finish cycling exercise in the heat. In contrast, the importance of carbohydrate supplementation appears to increase with longer TT efforts and/or in thermoneutral environments. To our knowledge, this study is the first to examine how push-to-the-finish cycling performance is affected by combinations of environmental conditions and rehydration with different beverages.

Physical performance is impaired in the heat compared to cooler conditions, attributable to greater cardiovascular strain caused by high skin and core temperatures (Rowell, 1974; Cheuvront et al., 2010). Further, exercise in the heat elicits a robust sweating response which, without proper fluid replacement, leads to dehydration. Dehydration ≥2% loss of body mass has been associated with acute deficits in physical performance (Pitts et al., 1944; Adolph, 1947; Maughan and Noakes, 1991; Below et al., 1995; Sawka et al., 2001; American College of Sports et al., 2007; Maughan and Shirreffs, 2010). Although ingestion of water may partially restore lost fluid, water alone is often not enough for achieving euhydration following exercise- and/or heat-induced dehydration. Ingestion of water alone reduces plasma osmolality, blunting the thirst response, subsequent fluid intake, and fluid retention, and thereby prolonging the time to restore plasma volume compared to beverages with added salt (Nose et al., 1988). Fluids with added components, especially salt or carbohydrates, can promote restoration of electrolytes and energy stores. These are often important constituents in the development of beverages for the purpose of promoting rehydration, such as sports drinks.

The components of a given beverage, namely fluid volume, energy content, and osmolality, can impact gastric emptying and absorption in the small intestine. In addition, the mineral constituents of these beverages are also metabolized on different time scales (Maughan, 1998). Thus, beverages with different compositions are likely to have varying effects on promoting hydration. This has led to increased interest in recent years regarding beverages, especially those that occur naturally, that may promote rehydration to a similar or greater extent than common sports drinks. Dairy-based beverages have a high electrolyte concentration (Maughan et al., 1997; Shirreffs and Maughan, 2000) and similar carbohydrate and caloric concentration to traditional CES beverages (Roy, 2008) and thus may serve as efficacious alternative fluid sources. Using the Beverage Hydration Index (BHI), Maughan et al. (2016) observed a similar hydration capacity of both skim milk and full-fat milk compared to an oral rehydration solution. Our laboratory more recently showed a novel beverage containing protein- and fat-free milk permeate (MPS), GoodSport^TM^, had a higher BHI than both water and a traditional carbohydrate-electrolyte drink (Gatorade®) in well-hydrated young subjects at rest (Berry et al., 2020). These findings were attributed to the greater electrolyte and osmolar constituents present in GS compared to the other beverages. However, it is important to note that those subjects were euhydrated at the onset of the study and sat at rest in thermoneutral conditions for the entirety of the study. It was previously unclear how these findings would translate to stressed conditions, such as following exercise in the heat, where dehydration is likely to occur. Based on the findings from the BHI study conducted by our lab, we hypothesized that consumption of the milk permeate beverage would promote rehydration to a greater extent than water or Gatorade following heat- and exercise-induced dehydration. However, in the current study, there were no differences in the amount of plasma volume expansion that occurred in all three drinking trials immediately following fluid consumption. There were also no differences in urine volume at any time point among the three drinking conditions, including after fluid consumption, indicating that fluid retention was similar across conditions. This suggests that in a hot environment following heat- and exercise-induced dehydration, fluid consumption either with or without added constituents similarly rehydrate young well-trained cyclists.

Previous literature indicates that during shorter bouts of maximal effort cycling in the heat, similar to that which may occur in the final kilometers of a cycling race, maintenance of hydration may be more important than carbohydrate ingestion. Adams et al. (2018) reported that during a maximum-effort 5-km cycling TT in the heat (35°C, 30% RH), fluid consumption following a 2-h dehydration period significantly improved average power output (295 W) compared to a trial in which no fluid replacement occurred (276 W), though fluid replacement occurred during exercise in this study rather than rehydration at rest prior to TT performance. Comparatively, Fan et al. (2020) observed no significant differences among trials in 20-km cycling TT performance in the heat (30°C, 75% RH) following exercise- and heat-induced dehydration following rehydration with water (39 min) or two carbohydrate-based beverages (sports drink: 38 min; oral rehydration solution: 38 min). These findings are similar to those of the current study in that rehydration alone improved maximum-effort push-to-the-finish cycling performance compared to a trial in which no fluid was consumed. In the current study, subjects were instructed to complete a 120-kJ TT, mimicking a push-to-the-finish race condition, in a hot-dry (35°C, 20% RH) environment. TT performance, measured as time-to-completion, absolute power output, and power output relative to body mass, was improved in each of the three trials in which fluid ingestion occurred compared to the no fluid condition (Figure 4), regardless of the type of fluid consumed.

A subset of four subjects repeated the three drinking conditions (WAT, GAT, and GS) to determine whether between-beverage differences in performance, attributable to carbohydrate load, were evident in a slightly longer TT occurring in thermoneutral conditions compared to the shorter TT in the heat. Interestingly, in a slightly prolonged TT (250 kJ) in thermoneutral conditions (21°C, 20% RH), average time-to-completion in GS (919 ± 353 s) was 41 s faster than WAT (960 ± 376 s) and 27 s faster than GAT (946 ± 365 s) (Figure 5). All four subjects in this prolonged TT in thermoneutral conditions improved their performance in the GS trial compared to WAT, while 3 of 4 subjects improved in the GAT trial compared to WAT. These findings indicate that different combinations of environmental conditions and time trial length may affect the influence and relative importance of carbohydrate supplementation vs. rehydration on cycling TT performance.

In the BHI study conducted by our lab (Berry et al., 2020), the elevated GS BHI was accompanied by a reduced free water clearance compared to both water and CES in the 2 h following fluid consumption, indicating that the kidneys were conserving more fluid following GS consumption relative to the other beverages. However, BHI studies are performed under standardized thermoneutral conditions with subjects at rest, i.e., renal function is unchallenged by the rigors of exercise and heat stress. During exercise in the heat, renal blood flow can be attenuated by as much as 50–60% and redistributed to skeletal muscle and skin for energetic and thermoregulatory purposes, respectively, thus reducing glomerular filtration rate and renal handling of ingested fluids (Ho et al., 1997). It was previously unknown how water, GS, or GAT may differentially impact hydration status and subsequent push-to-the-finish exercise performance in either a thermoneutral environment or hot-dry environment, in which renal blood flow, GFR, and subsequent fluid filtration and retention would be challenged. Although kidney function was not directly measured in the present study, we observed similar progressive reductions in estimated glomerular filtration rate, a proxy measurement for kidney function, across all time-points in each drink condition in the hot-dry environment. It is plausible that the reduction in renal blood flow and subsequent attenuation of eGFR observed in the hot condition utilized in the present study offset the beneficial effects of beverages containing carbohydrates and electrolytes, while such beneficial effects may be preserved in thermoneutral conditions. Thus, it is likely that during a shorter time-trial in hot-dry conditions, maintenance of hydration is more important for push-to-the-finish cycling performance than carbohydrate supplementation, whereas carbohydrate supplementation exerts a greater influence in thermoneutral conditions and/or during push-to-the-finish tasks. Future research should examine how performance is impacted across a wider range of combinations of environments and time-trial lengths.

### Limitations

Although all subjects were similarly dehydrated (~2%) following the first bout of cycling and remained similarly dehydrated in the three beverage consumption conditions at each time-point for the remainder of the study, it is possible the subjects were dehydrated to a greater extent than actually observed throughout the study. Body water volume and serum osmolality are tightly regulated such that osmolality is maintained within a range of 275–295 mosm·kg^−1^ under euhydrated conditions. Reductions in body water content without proper replacement can stimulate increases in serum osmolality beyond the upper limit of euhydration of 295 mosm·kg^−1^. Interestingly, subjects in the current study on average had a baseline plasma osmolality of 297–298 mosm·kg^−1^, indicating subjects were dehydrated, despite having normal serum electrolyte concentrations and urine specific gravity (1.009–1.010) values on average. In a previous study in our lab examining the BHI of a novel milk-permeate solution similar to the one utilized in the current study, baseline serum osmolality was 291 mosm·kg^−1^, within the normal range of euhydration (Berry et al., 2020). In both studies, subjects were instructed to maintain their normal hydration habits in the 24 h prior to each trial, and were given a 500 ml bottle of water to consume 1 h before each trial to ensure euhydration. An overnight fast was also completed prior to each trial in both studies. However, in the current study, subjects were provided a carbohydrate energy bar to consume along with 500 ml water. This resulted in an increased serum glucose concentration in the current study compared to the previous study, which may in part help explain the further elevation in serum osmolality in the current study compared to the prior BHI study.

Following REH, plasma glucose concentrations were significantly elevated in the GS trial compared to WAT and NF, and in the GAT trial compared to all other conditions. Comparatively, there were no differences in plasma glucose concentrations among the three trials in which a beverage was consumed. Considering the relatively high variance in time trial time-to-completion among subjects, it is possible that the time-course of changes in plasma glucose concentrations may have been different between individual subjects. However, given that the time between collection of the post-REH and post-TT blood samples was <1 h for all subjects in all trials, it is unlikely that any subject in the hot-dry conditions was sufficiently glucose/glycogen depleted such that the time-course of these glucose responses would differently impact performance among subjects. Further, during short bouts of exercise at a high-intensity (>85% VO_2max_), oxidation of stored muscle glycogen contributes a large majority (~67%) of the necessary substrate for energy production (Coyle, 1995). Plasma glucose supplies only a small contribution during short bouts of exercise at intensities >85% VO_2max_ when stored muscle glycogen is abundant, but can supply upwards of 40% of energy once stored muscle glycogen is sufficiently depleted (Coyle, 1995). However, it is unlikely that subjects were glycogen depleted prior to the TT. Subjects cycled at a moderate intensity for ~60 min during the dehydration period, and an additional 30 min during the warm-up period at a light intensity. At moderate intensities in endurance-trained subjects, glycogen depletion usually occurs approximately anywhere from 1 to 3 h after onset of exercise (Coyle, 1995). It is possible that in the current study, subjects were not glycogen depleted, and plasma glucose derived from ingestion of the two beverages containing carbohydrates (GAT: 82 ± 16 g sugar consumed; GS: 54 ± 10 g sugar consumed) may have served a minimal role in substrate utilization during the TT. Given that the three drink conditions in the hot dry condition all improved TT performance, but were not different from each other, it is conceivable that hydration status, rather than carbohydrate provision, exerts a greater influence on performance during short bouts of high-intensity exercise in the heat. In contrast, in the thermoneutral condition, it is possible that the slightly extended TT resulted in further muscle glycogen depletion and a shift toward a greater reliance on plasma glucose concentrations for energy production. Thus, carbohydrate supplementation, rather than hydration status, may play a larger influential role on athletic performance during slightly longer push-to-the-finish exercise in thermoneutral conditions.

Subjects remained clothed during each measurement of body mass. While the clothing may have retained some sweat during the cycling periods, limiting the reduction in measured body mass, the dry environmental conditions mitigated retention. Had we assessed nude body mass, subjects would have had to undress for every measurement, extending time spent off the bicycle during cycling sessions and adding to subject burden. Similar methodology has been used in dehydration studies involving cycling (Adams et al., 2018, 2019), and given the cross-over nature of the experimental design, it is unlikely that this limitation had differential effects on specific drink condition trials.

In the current study, rehydration occurred over a 30-min period immediately following an exercise- and heat-induced dehydration of ~2% loss of body mass. However, under normal circumstances, it is likely that thirst sensation would be stimulated before reaching such a degree of dehydration (Shirreffs et al., 2004). Indeed, thirst sensation (measured as a 1–9 scale, 1 = “not thirsty at all,” 9 = “very, very thirsty”) was similarly elevated in each of the four trials compared to baseline (NF = 7 ± 2, WAT = 8 ± 1, GAT = 8 ± 1, GS = 8 ± 1), indicating that subjects on average were “very thirsty” following dehydration. Planned drinking strategies or *ad libitum* fluid consumption are often implemented during road cycling sessions. Kenefick stated that planned drinking is optimal during endurance exercise lasting >90 min, particularly when it is occurring in the heat, while drinking to thirst or *ad libitum* drinking may be more optimal during short duration exercise lasting <60–90 min (Kenefick, 2018). Future investigation is warranted regarding how planned and/or *ad libitum* drinking of a beverage containing ultra-filtered deproteinized milk vs. a traditional carbohydrate-based beverage may differentially promote rehydration or maintenance of euhydration during exercise, rather than after exercise has ceased and subjects are already dehydrated, as well as how this may subsequently impact athletic performance.

## Conclusion

The present study demonstrated that fluid consumption following exercise- and heat-induced dehydration improves high-intensity cycling time-trial performance in young well—trained cyclists. However, neither a beverage containing ultra-filtered deproteinized milk that is high in electrolytes nor a traditional carbohydrate-electrolyte sports drink further improved performance in this short push-to-the-finish type bout compared to water. In a subset of four subjects, cycling performance was improved in a slightly longer time-trial in thermoneutral conditions following consumption of either GS or GAT compared to water. These findings suggest that hydration is more important than carbohydrate provision during shorter push-to-the-finish cycling in the heat. However, carbohydrate provision plays an emerging role in slightly longer push-to-the-finish cycling in thermoneutral conditions. Future investigation is necessary to elucidate whether these differences in performance between beverage and environmental conditions persist with longer periods of exercise (i.e. >1-h in length) and across a wider range of combinations of environmental conditions and push-to-the-finish target length.

## Data Availability Statement

The raw data supporting the conclusions of this article will be made available by the authors, without undue reservation.

## Ethics Statement

The studies involving human participants were reviewed and approved by Pennsylvania State University Institutional Review Board. The patients/participants provided their written informed consent to participate in this study.

## Author Contributions

WK: conceptualization, supervision, and project administration. CB, SW, and WK: methodology. CB, SW, RC, and WK: formal analysis and writing—review and editing. CB, SW, and RC: investigation. CB: data curation and writing—original draft preparation. All authors have read and agreed to the published version of the manuscript.

## Funding

This research was supported by funding from the BUILD Dairy Program, Western Dairy Center.

## Conflict of Interest

The authors declare that the research was conducted in the absence of any commercial or financial relationships that could be construed as a potential conflict of interest.

## Publisher's Note

All claims expressed in this article are solely those of the authors and do not necessarily represent those of their affiliated organizations, or those of the publisher, the editors and the reviewers. Any product that may be evaluated in this article, or claim that may be made by its manufacturer, is not guaranteed or endorsed by the publisher.

## References

[B1] Adams J. Scott D. M. Brand N. A. Suh H. G. Seal A. D. McDermott B. P.. (2019). Mild hypohydration impairs cycle ergometry performance in the heat: a blinded study. Scand. J. Med. Sci. Sports 29, 686–695. 10.1111/sms.13386 30659665

[B2] Adams J. D. Sekiguchi Y. Suh H. G. Seal A. D. Sprong C. A. Kirkland T. W.. (2018). Dehydration impairs cycling performance, independently of thirst: a blinded study. Med. Sci. Sports Exerc. 50, 1697–1703. 10.1249/MSS.0000000000001597 29509643

[B3] Adolph E. F. (1947). Physiology of Man in the Desert. New York and London: Interscience Publishers, Inc.

[B4] American College of Sports M. Sawka M. N. Burke L. M. Eichner E. R. Maughan R. J. Montain S. J.. (2007). American college of sports medicine position stand. Exercise and fluid replacement. Med. Sci. Sports Exerc. 39, 377–390. 10.1249/mss.0b013e31802ca597 17277604

[B5] Arnaoutis G. Kavouras S. A. Angelopoulou A. Skoulariki C. Bismpikou S. Mourtakos S.. (2015). Fluid balance during training in elite young athletes of different sports. J. Strength Cond. Res. 29, 3447–3452. 10.1519/JSC.0000000000000400 24513625 PMC4515206

[B6] Below P. R. Mora-Rodriguez R. Gonzalez-Alonso J. Coyle E. F. (1995). Fluid and carbohydrate ingestion independently improve performance during 1 h of intense exercise. Med. Sci. Sports Exerc. 27, 200–210. 10.1249/00005768-199502000-00009 7723643

[B7] Berry C. W. Wolf S. T. Murray B. Kenney W. L. (2020). Hydration efficacy of a milk permeate-based oral hydration solution. Nutrients 12:1502. 10.3390/nu12051502 32455677 PMC7284605

[B8] Cheuvront S. N. Kenefick R. W. Montain S. J. Sawka M. N. (2010). Mechanisms of aerobic performance impairment with heat stress and dehydration. J. Appl. Physiol. 109, 1989–1995. 10.1152/japplphysiol.00367.2010 20689090

[B9] Coyle E. F. (1995). Substrate utilization during exercise in active people. Am. J. Clin. Nutr. 61(4 Suppl), 968S–979S. 10.1093/ajcn/61.4.968S 7900696

[B10] Coyle E. F. (2004). Fluid and fuel intake during exercise. J. Sports Sci. 22, 39–55. 10.1080/0264041031000140545 14971432

[B11] Dill D. B. Costill D. L. (1974). Calculation of percentage changes in volumes of blood, plasma, and red cells in dehydration. J. Appl. Physiol. 37, 247–248. 10.1152/jappl.1974.37.2.247 4850854

[B12] Fan P. W. Burns S. F. Lee J. K. W. (2020). Efficacy of ingesting an oral rehydration solution after exercise on fluid balance and endurance performance. Nutrients 12:3826. 10.3390/nu12123826 33333771 PMC7765193

[B13] Gisolfi C. V. Lambert G. P. Summers R. W. (2001). Intestinal fluid absorption during exercise: role of sport drink osmolality and [Na+]. Med. Sci. Sports Exerc. 33, 907–915. 10.1097/00005768-200106000-00009 11404655

[B14] González-Alonso J. Calbet J. A. Nielsen B. (1998). Muscle blood flow is reduced with dehydration during prolonged exercise in humans. J. Physiol. 513, 895–905. 10.1111/j.1469-7793.1998.895ba.x 9824726 PMC2231307

[B15] Ho C. W. Beard J. L. Farrell P. A. Minson C. T. Kenney W. L. (1997). Age, fitness, and regional blood flow during exercise in the heat. J. Appl. Physiol. 82, 1126–1135. 10.1152/jappl.1997.82.4.1126 9104849

[B16] Jeukendrup A. E. (2004). Carbohydrate intake during exercise and performance. Nutrition 20, 669–677. 10.1016/j.nut.2004.04.017 15212750

[B17] Kenefick R. W. (2018). Drinking strategies: planned drinking versus drinking to thirst. Sports Med. 48(Suppl 1), 31–37. 10.1007/s40279-017-0844-6 29368181 PMC5790864

[B18] Magee P. J. Gallagher A. M. McCormack J. M. (2017). High prevalence of dehydration and inadequate nutritional knowledge among university and club level athletes. Int. J. Sport Nutr. Exerc. Metab. 27, 158–168. 10.1123/ijsnem.2016-0053 27710146

[B19] Marriott B. M. (1993). “Water requirements during exercise in the heat,” in Nutritional Needs in Hot Environments: Applications for Military Personnel in Field Operations. Washington, DC: National Academies Press. 25144014

[B20] Maughan R. (1998). The sports drink as a functional food: formulations for successful performance. Proc. Nutr. Soc. 57, 15–23. 10.1079/PNS19980005 9571704

[B21] Maughan R. J. Leiper J. B. Shirreffs S. M. (1997). Factors influencing the restoration of fluid and electrolyte balance after exercise in the heat. Br. J. Sports Med. 31, 175–182. 10.1136/bjsm.31.3.175 9298549 PMC1332513

[B22] Maughan R. J. McArthur M. Shirreffs S. M. (1996). Influence of menstrual status on fluid replacement after exercise induced dehydration in healthy young women. Br. J. Sports Med. 30, 41–47. 10.1136/bjsm.30.1.41 8665117 PMC1332264

[B23] Maughan R. J. Noakes T. D. (1991). Fluid replacement and exercise stress. A brief review of studies on fluid replacement and some guidelines for the athlete. Sports Med. 12, 16–31. 10.2165/00007256-199112010-00003 1925187

[B24] Maughan R. J. Shirreffs S. M. (2010). Dehydration and rehydration in competative sport. Scand. J. Med. Sci. Sports 20 (Suppl 3), 40–47. 10.1111/j.1600-0838.2010.01207.x 21029189

[B25] Maughan R. J. Watson P. Cordery P. A. Walsh N. P. Oliver S. J. Dolci A.. (2016). A randomized trial to assess the potential of different beverages to affect hydration status: development of a beverage hydration index. Am. J. Clin. Nutr. 103, 717–723. 10.3945/ajcn.115.114769 26702122

[B26] Menaspà P. Quod M. Martin D. Peiffer J. Abbiss C. J. I. (2015). Physical demands of sprinting in professional road cycling. 36, 1058–1062. 10.1055/s-0035-1554697 26252551

[B27] Murray B. (2007). Hydration and physical performance. J. Am. Coll. Nutr. 26(5 Suppl), 542S–548S. 10.1080/07315724.2007.10719656 17921463

[B28] Nielsen B. Sjogaard G. Ugelvig J. Knudsen B. Dohlmann B. (1986). Fluid balance in exercise dehydration and rehydration with different glucose-electrolyte drinks. Eur. J. Appl. Physiol. Occup. Physiol. 55, 318–325. 10.1007/BF02343806 3732259

[B29] Nose H. Mack G. W. Shi X. R. Nadel E. R. (1988). Role of osmolality and plasma volume during rehydration in humans. J. Appl. Physiol. 65, 325–331. 10.1152/jappl.1988.65.1.325 3403476

[B30] Orru S. Imperlini E. Nigro E. Alfieri A. Cevenini A. Polito R.. (2018). Role of functional beverages on sport performance and recovery. Nutrients 10:1470. 10.3390/nu10101470 30308976 PMC6213308

[B31] Passe D. H. Horn M. Murray R. (2000). Impact of beverage acceptability on fluid intake during exercise. Appetite 35, 219–229. 10.1006/appe.2000.0352 11073704

[B32] Pitts G. Johnson R. Consolazio F. (1944). Work in the heat as affected by intake of water, salt and glucose. Am. J. Physiol. Legacy Content 142, 253–259. 10.1152/ajplegacy.1944.142.2.253

[B33] Rivera-Brown A. M. Gutierrez R. Gutierrez J. C. Frontera W. R. Bar-Or O. (1999). Drink composition, voluntary drinking, and fluid balance in exercising, trained, heat-acclimatized boys. J. Appl. Physiol. 86, 78–84. 10.1152/jappl.1999.86.1.78 9887116

[B34] Rowell L. B. (1974). Human cardiovascular adjustments to exercise and thermal stress. Physiol. Rev. 54, 75–159. 10.1152/physrev.1974.54.1.75 4587247

[B35] Roy B. D. (2008). Milk: the new sports drink? a review. J. Int. Soc. Sports Nutr. 5:15. 10.1186/1550-2783-5-15 18831752 PMC2569005

[B36] Sawka M. N. Montain S. J. Latzka W. A. (2001). Hydration effects on thermoregulation and performance in the heat. Comp. Biochem. Physiol. Part A Mol. Integr. Physiol. 128, 679–690. 10.1016/S1095-6433(01)00274-4 11282312

[B37] Shirreffs S. (2009). Hydration in sport and exercise: water, sports drinks and other drinks. Nutr. Bull. 34, 374–379. 10.1111/j.1467-3010.2009.01790.x

[B38] Shirreffs S. M. Aragon-Vargas L. F. Keil M. Love T. D. Phillips S. (2007). Rehydration after exercise in the heat: a comparison of 4 commonly used drinks. Int. J. Sport Nutr. Exerc. Metab. 17, 244–258. 10.1123/ijsnem.17.3.244 17693686

[B39] Shirreffs S. M. Maughan R. J. (2000). Rehydration and recovery of fluid balance after exercise. Exerc. Sport Sci. Rev. 28, 27–32. 11131686

[B40] Shirreffs S. M. Merson S. J. Fraser S. M. Archer D. T. (2004). The effects of fluid restriction on hydration status and subjective feelings in man. Br. J. Nutr. 91, 951–958. 10.1079/BJN20041149 15182398

[B41] Shirreffs S. M. Taylor A. J. Leiper J. B. Maughan R. J. (1996). Post-exercise rehydration in man: effects of volume consumed and drink sodium content. Med. Sci. Sports Exerc. 28, 1260–1271. 10.1097/00005768-199610000-00009 8897383

[B42] Temesi J. Johnson N. A. Raymond J. Burdon C. A. O'Connor H. T. (2011). Carbohydrate ingestion during endurance exercise improves performance in adults. J. Nutr. 141, 890–897. 10.3945/jn.110.137075 21411610

[B43] Wilk B. Bar-Or O. (1996). Effect of drink flavor and NaCL on voluntary drinking and hydration in boys exercising in the heat. J. Appl. Physiol. 80, 1112–1117. 10.1152/jappl.1996.80.4.1112 8926234

